# A role for RNA post-transcriptional regulation in satellite cell activation

**DOI:** 10.1186/2044-5040-2-21

**Published:** 2012-10-09

**Authors:** Nicholas H Farina, Melissa Hausburg, Nicole Dalla Betta, Crystal Pulliam, Deepak Srivastava, DDW Cornelison, Bradley B Olwin

**Affiliations:** 1 Department of Molecular, Cellular, and Developmental Biology, University of Colorado, Boulder, CO, 80309, USA; 2 Laboratory of Signal Transduction, National Institute of Environmental Health Sciences, Research Triangle Park, Triangle Park, NC, 22709, USA; 3 Gladstone Institute of Cardiovascular Disease, University of San Francisco, San Francisco, CA, 94158, USA; 4 Biological Sciences and Bond Life Sciences Center, University of Missouri, Columbia, MO, 65211, USA

**Keywords:** Satellite cell, RNA post-transcriptional regulation, microRNA.

## Abstract

**Background:**

Satellite cells are resident skeletal muscle stem cells responsible for muscle maintenance and repair. In resting muscle, satellite cells are maintained in a quiescent state. Satellite cell activation induces the myogenic commitment factor, MyoD, and cell cycle entry to facilitate transition to a population of proliferating myoblasts that eventually exit the cycle and regenerate muscle tissue. The molecular mechanism involved in the transition of a quiescent satellite cell to a transit-amplifying myoblast is poorly understood.

**Methods:**

Satellite cells isolated by FACS from uninjured skeletal muscle and 12 h post-muscle injury from wild type and Syndecan-4 null mice were probed using Affymetrix 430v2 gene chips and analyzed by Spotfire^tm^ and Ingenuity Pathway analysis to identify gene expression changes and networks associated with satellite cell activation, respectively. Additional analyses of target genes identify miRNAs exhibiting dynamic changes in expression during satellite cell activation. The function of the miRNAs was assessed using miRIDIAN hairpin inhibitors.

**Results:**

An unbiased gene expression screen identified over 4,000 genes differentially expressed in satellite cells *in vivo* within 12 h following muscle damage and more than 50% of these decrease dramatically. RNA binding proteins and genes involved in post-transcriptional regulation were significantly over-represented whereas splicing factors were preferentially downregulated and mRNA stability genes preferentially upregulated. Furthermore, six computationally identified miRNAs demonstrated novel expression through muscle regeneration and in satellite cells. Three of the six miRNAs were found to regulate satellite cell fate.

**Conclusions:**

The quiescent satellite cell is actively maintained in a state poised to activate in response to external signals. Satellite cell activation appears to be regulated by post-transcriptional gene regulation.

## Background

Skeletal muscle is terminally differentiated and thus, requires a population of resident adult stem cells, satellite cells, for maintenance and repair [1-3]. Satellite cells are typically mitotically quiescent in resting muscle and activate to prepare for cell cycle entry by HGF [4,5], nitric oxide [6], and TNFα [7], upon a muscle injury. Intracellular p38α/β MAPK and downstream signaling is stimulated upon satellite cell activation, permitting MyoD induction (Troy *et al.*)^a^[8], S-phase entry [8,9], and subsequent proliferation. A subset of satellite cells self-renew to maintain the satellite cell pool (Troy *et al.*)^a^[10,11] and generate a rapidly proliferating transit-amplifying myoblast population (Troy *et al*.)^a^[10].

The transition from a quiescent satellite cell to a proliferating, transit amplifying myoblast was thought to require extensive transcriptional induction as quiescent satellite cells have a low ratio of cytoplasmic volume to nuclear volume, few cellular organelles, tightly packed heterochromatin, and are believed to be metabolically inactive [12,13]. However, recent evidence suggests that satellite cell quiescence is ‘active’ and satellite cells are poised to react to external stimuli after muscle damage [14]. Moreover, quiescent fibroblasts exhibit high metabolic activity [15] in agreement with a quiescent state that is far from ‘quiet’. Interestingly, a growing pool of data demonstrates that cell fate determination is reliant on post-transcriptional gene regulation [16-20] and may provide mechanisms to maintain quiescent satellite cells in a ready state.

One such RNA post-transcriptional mechanism, microRNA-mediated gene silencing, regulates skeletal muscle specification and myogenic differentiation [21-23]. MicroRNAs (miRNA) are a class of small non-coding RNAs that bind to target mRNA in a sequence specific manner to mediate gene silencing [24-27] and can target and silence protein expression from tens to hundreds of mRNAs [26,27]. Furthermore, miRNAs modulate stem cell fate decisions [28-31] and may have similar functions in satellite cells. Recent studies identify miR-489 and miR-206 expression in quiescent satellite cells [32,33], however, it is likely that many uncharacterized miRNAs play roles in the transition of a quiescent satellite cell to transit-amplifying myoblast.

To understand the mechanisms involved in satellite cell activation, we previously screened a number of candidate genes for changes in expression from freshly isolated satellite cells and from satellite cells isolated at either 12 h post-muscle injury or 48 h post-muscle injury to represent quiescent, activated, and proliferating satellite cells, respectively. Although unbiased gene expression screens have been performed on satellite cells, these studies have either compared freshly isolated satellite cells to satellite cells expanded in culture [14,34] or to satellite cells in diseased skeletal muscle [14]. Neither of these studies directly compared satellite cells prior to and following induced muscle injury *in vivo* and thus, the reported gene expression changes specific to cell culture or specific to diseased muscle may not reliably identify gene expression changes associated with satellite cell activation *in vivo*. Here, we report global gene expression profiles and candidate miRNAs associated with quiescent and activated satellite cells as well as identify a novel function for miR-16, miR-106b, and miR-124 in satellite cell fate determination. From these analyses, we posit that satellite cell activation is primarily regulated by post-transcriptional gene regulation as opposed to transcriptional induction.

## Methods

### Mice

All animal procedures were performed according to protocol number 1012.01 approved by Institutional Animal Care and Use Committee at the University of Colorado at Boulder. Mice were housed in a pathogen-free environment at the University of Colorado at Boulder. All mice sacrificed were female and between 3 and 6 months of age. Wild type mice were C57Bl/6xDBA2 (B6D2F1/J, Jackson Labs) and syndecan-4^−/−^ mice carry homozygous deletion of syndecan-4 in the C57Bl/6 background [35].

### Fluorescence-activated cell sorting of satellite cells

The tibialis anterior muscles of 3-month-old female B6D2F1/J or syndecan-4^−/−^ mice were injured by injection with 50 μL 1.2% BaCl_2_ in saline prior to harvest or harvested from uninjured hind limbs. The tibialis anterior muscles were dissected from the hind limb, minced, and digested in 400 U/mL collagenase in Ham’s F-12C at 37°C for 1 h, vortexing frequently. Collagenase was inactivated by the addition of horse serum and debris was removed by sequential straining through 70 μm and 40 μm cell strainers (BD Falcon). Cells were gently centrifuged and the cell pellets were incubated at 4°C with 1:100 rabbit anti-syndecan-3 antibody in Ham’s F-12C with 15% horse serum followed by an incubation on ice with Cy5 conjugated anti-rabbit-IgG (Molecular Probes). Satellite cells were sorted based on syndecan-3 immunoreactivity on a MoFlo Legacy cell sorter (Dako Cytomation) directly into RNA lysis buffer (PicoPure RNA Isolation kit, Arcturus).

### Myofiber explant culture and immunostaining

All hind limb muscles were dissected, connective tissue removed, and individual muscle groups isolated followed by digestion in 400 U/mL collagenase in Ham’s F-12C at 37°C. Single myofibers were isolated and grown in Ham’s F-12C supplemented with 15% horse serum and 0.5 nM FGF-2 prior to fixation in 4% PFA. Fibers were blocked in 10% normal goat serum in phosphate buffered saline followed by antibody staining. Primary antibodies were rabbit anti-cmet (Santa Cruz) at 1:100, mouse anti-MyoD (Novocastra) at 1:10, mouse anti-Pax7 at 1:5 (Developmental Studies Hybridoma Bank), and rabbit anti-MyoD C-20 at 1:500 (Santa Cruz Biotechnology). Secondary antibodies were Alexa-488 conjugated anti-mouse IgG, Alexa-594 conjugated anti-rabbit IgG, Alexa-555 conjugated anti-mouse IgG, and Alexa-647 conjugated anti-rabbit IgG (Molecular Probes). All images taken on a Nikon Eclipse E800 microscope with a Nikon 40x/0.75 differential interference contrast M lens and analyzed with Slidebook (Intelligent Imaging Innovations, Inc.).

### Microarray hybridization

RNA was isolated from satellite cells using the PicoPure RNA Isolation kit (Arcturus) followed by two rounds of linear T7-based amplification (RiboAmp HA kit: Arcturus). The RNA equivalent of 5,000 cells was hybridized to Affymetrix mouse 430v2 GeneGhips (MOE430v2) according to manufacturer’s instructions. GeneChips were scanned at the University of Colorado at Boulder on an Affymetrix GeneChip Scanner 3000 and spot intensities were recovered in the GeneChip Operating System (Affymetrix).

### Microarray data processing and analysis

All analysis was performed using Spotfire™ DecisionSite 2 for Microarray Analysis. The raw CEL data files were normalized using GC Robust Multi-array Analysis (GCRMA). The raw CEL data files, microarray metadata, and GCRMA normalized expression values were deposited in GEO datasets (GSE38870). One wild type freshly isolated satellite cell replicate consistently clustered with the wild type satellite cells 12 h post-injury replicates (via hierarchical, Self-Organizing Map (SOM), k-means) indicating myogenic commitment and was removed from our analysis. The hierarchical cluster and associated dendrogram were generated using the log_2_-value for relative probe intensity using the Unweighted Pair Group Method with Arithmetic Mean (UPGMA) with Euclidean distance as the similarity measure. The significance between genotypes and time points was determined using the multifactor analysis of variance (ANOVA) with a false discovery rate (FDR) ≤ 0.05 and Bonferroni adjustment. Fold change was calculated as 2^abs(difference)^, where difference is the log_2_ difference between samples compared. Venn diagrams were generated in Spotfire™ using the list comparison function.

### Gene ontology and biological pathway analysis

Unique gene identifiers (gene symbol, entrez gene ID, or Affymetrix probeset ID) were uploaded to the Database for Annotation, Visualization and Integrated Discovery (DAVID http://david.abcc.ncifcrf.gov/), FunNet (http://www.funnet.info/), and ProfCom (http://webclu.bio.wzw.tum.de/profcom/). The mouse genome reference dataset for each algorithm was used as background. No further settings were required for DAVID. Analysis setting for FunNet were conventional functional analysis with the specificity enrichment computation for GO and false discovery rate ≤ 5%. Analysis settings for ProfCom were up degree 1 and exclude. The default settings for each algorithm were used to identify enriched GO terms. Affymetrix probeset IDs and the log_2_ difference between wild type freshly isolated satellite cells and satellite cells isolated 12 h post-injury were uploaded and analyzed using IPA v9.0 (Ingenuity® Systems http://www.ingenuity.com). Analysis settings were to consider both all direct and indirect molecules and/or relationships using the Mouse Genome 430 2.0 Array as a reference dataset.

### Computational prediction of miRNAs

The Srivastava lab algorithm assessed mRNA sequences in human, mouse, and rat for miRNA seed matches and required base-paring of miRNA nucleotides 2 to 7 with binding energy ≤ −14 kcal/mol and flanking energy ≥ −7 kcal/mol. Secondary structure was used to eliminate false-positives by removing those seed matches with secondary elements that stabilize mRNA. Priority 1 calls had a destabilizing mRNA element while Priority 2 calls did not contain a destabilizing element in at least one species. GeneAct used the miRanda algorithm to identify miRNA target sites across three mammalian species. False-positives were eliminated with the differential binding site search against genes that were constitutively expressed in satellite cells.

### RNA isolation

mRNA was extracted from satellite cells using the RNeasy Kit according to the manufacturer’s protocol (Qiagen). miRNA was extracted using both the RNAqueous-micro kit the mirVana miRNA isolation kit according to the manufacturer’s protocols (Ambion) with the following modifications. For satellite cells, the mirVana manufacturer’s protocol was followed using volumes and columns for the RNAqueous-micro kit. For MM14 cells, the mirVana manufacturer’s protocol was followed. RNA concentration was determined using a NanoDrop 2000 spectrophotometer (Thermo Scientific).

### Quantitative RT-PCR

The Superscript III First Strand cDNA Synthesis kit was used to generate cDNA from mRNA according to manufacturer’s instructions (Invitrogen). The Ncode miRNA qRT-PCR system (Invitrogen) was used to generate cDNA from miRNA according to manufacturer’s instructions. Briefly, RNA was poly-adenylated with Poly-A polymerase followed by cDNA transcription with Superscript III reverse transcriptase using a primer similar to Oligo-dT with a unique 5’ end. Quantitative RT-PCR was performed using SYBR-Green (Applied Biosystems) or SYBR-GreenER (Invitrogen) on either an ABI 7500 Fast or ABI 7900 Real-Time PCR machine (Applied Biosystems). Primer sequences are listed in Table 1.

**Table 1:** Quantitive PCR primer sequences

**Gene**	**Primer type**	**Sequence**
GAPDH	Forward	5’ - TGTGTCCGTCGTGGATCTGA - 3’
	Reverse	5’ - CCTGCTTCACCACCTTCTTGA - 3’
18S	Forward	5’ - GCCGCTAGAGGTGAAATTCTTG - 3’
	Reverse	5’ - CTTTCGCTCTGGTCCGTCTT - 3’
Celf4	Forward	5’ CCTGCTCATCTACCATCTGCC - 3’
	Reverse	5’ - GCTCACGAAGCCAAAGCATTT - 3’
Pabpn1	Forward	5’ - TTTCCTTGCCCTGTTTCCCATGTC - 3’
	Reverse	5’ - AGTGACTGAAGGGAGCACCTCAAA - 3’
Ppargc1a	Forward	5’ - TAGTTTGAGCCCTTGCTGGCTCTT - 3’
	Reverse	5’ - AGCTCAGTGAGGCTGATGTGTACT - 3’
Mbnl1	Forward	5’ - AACTGGACAGAACCGGGAAGAACT - 3’
	Reverse	5’ - GCAAACTGCAACTTGTGACACGGA - 3’
Matr3	Forward	5’ - ATTGTGGATAGGGCCAGTCATGGT - 3’
	Reverse	5’ - TTGCATTTGAGACAAGTGGCCTGG - 3’
Sfrs3	Forward	5’ - TGTGGCACTGTGGGTGGAATGATA - 3’
	Reverse	5’ - CTGAAAGGACACTGGCATCTGAGT - 3’
Zfp36	Forward	5’ - TCTCTGCCATCTACGAGAGCC - 3’
	Reverse	5’ - CCAGTCAGGCGAGAGGTGA - 3’
Zfp36l1	Forward	5’ - GCTTTCGAGACCGCTCTTTCT - 3’
	Reverse	5’ - TTGTCCCCGTACTTACAGGCA - 3’
Zfp36l2	Forward	5’ - AGCGGCTCCCAGATCAACT - 3’
	Reverse	5’ - CGAAAGCGAAGGCGTTGTTA - 3’
Elavl1	Forward	5’ - TGTGAGTCACCAGCTGCCAAGTAT - 3’
	Reverse	5’ - GAGGTGGTTCAAACCAACCAACCA - 3’
Cdk2	Forward	5’ - TCCTCTGAGAGCAGTGATGCA - 3’
	Reverse	5’ - TTCCCCCAATGACCTAACCAG - 3’
E2F3	Forward	5’ - GGTCCTGGATCTGAACAAGGC - 3’
	Reverse	5’ - CCTTCCAGCACGTTGGTGAT - 3’
U6	Reverse	5’ - AATTCGTGAAGCGTTCCATAT - 3’
miR-16	Reverse	5’ - TAGCAGCACGTAAATATTGGCG - 3’
miR-93	Reverse	5’ - CAAAGTGCTGTTCGTGCAGGTAG - 3’
miR-106b	Reverse	5’ - TAAAGTGCTGACAGTGCAGAT - 3’
miR-107	Reverse	5’ - AGCAGCATTGTACAGGGCTATCA - 3’
miR-124	Reverse	5’ - TAAGGCACGCGGTGAATGCC - 3’
miR-200b	Reverse	5’ - TAATACTGCCTGGTAATGATGA - 3’

Sequences for forward and reverse primers used to detect mRNAs or sequence of reverse primer used to detect miRNAs using the Ncode universal forward primer.

### RNase protection assay

Candidate miRNAs were screened using the mirVana miRNA detection kit according to the manufacturer’s protocol (Ambion). All probes were radio-labeled with ^32^P-UTP using the mirVana miRNA probe construction kit (Ambion) according to manufacturer’s protocol.

### Isolation of quiescent satellite cells

Quiescent satellite cells were isolated following IP injection with 75 mg/kg of SB203580 (Alexis Corporation) and kept in 25 μM SB203580 through the isolation as described (Hausburg *et al*., Submitted). All satellite cells were isolated as follows. Hind limb muscles of 3- to 6-month-old female B6D2F1/J mice were dissected and digested in 400 U/mL collagenase in Ham’s F-12C at 37°C for 1 h with periodic vortexing. The collagenase was inactivated with horse serum and debris was removed with sequential straining through 70 μm and 40 μm cell strainers (BD Falcon). Satellite cells were either isolated at the interface of a 40%/70% percoll gradient (GE Healthcare) or plated in Ham’s F-12C supplemented with 15% horse serum and 0.5nM FGF-2 for various times before RNA isolation.

### miRNA inhibition

Myofiber explant cultures were transfected using Lipofectamine 2000 (Invitrogen) according to manufacturer’s protocol with a 2.5:1 ratio of lipofectamine:nucleic acid. 200nM miRIDIAN hairpin inhibitors (Dharmacon) against miR-16, miR-93, miR-106b, and miR-124 were co-transfected with pEGFP-C1-H2B. Scrambled control was the miRIDIAN hairpin inhibitor negative control 1 (Dharmacon).

## Results

### Identification of gene expression changes associated with satellite cell activation *in vivo*

**Figure 1:**
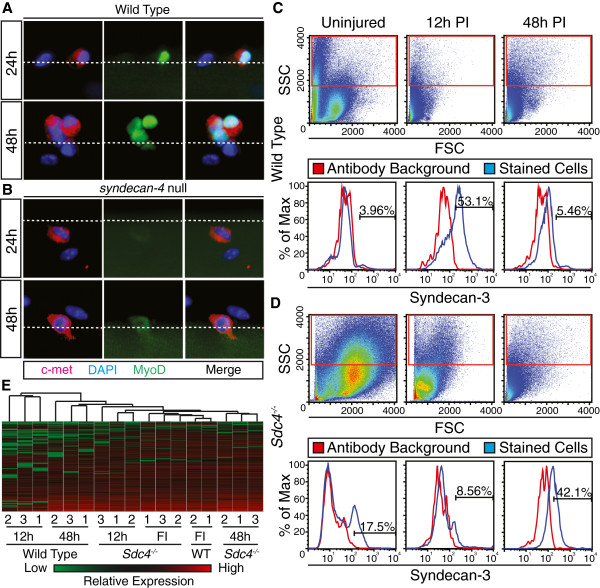
**Sdc4**^**−/− **^**satellite cell gene expression post-muscle injury is similar to freshly isolated satellite cells.** Myofiber-associated satellite cells are immunoreactive for MyoD 24 h and 48 h after isolation from wild type mice but not Sdc4^−/−^ mice (**A**, **B**). Wild type but not Sdc4^−/−^ cells divide by 48 h in culture (**B**) where c-met (red), MyoD (green), and DAPI (blue) identify satellite cells and a dashed line indicates the position of the myofiber membrane (**A**, **B**). Flow cytometry histograms of wild type (**C**) and syndecan-4 null (**D**) mononuclear cells from uninjured and injured skeletal muscle 12 h and 48 h post-injury plotted for cell size (FSC) *vs.* internal complexity (SSC), where the red box indicates gating for further analysis to remove debris (upper panels). Syndecan-3 immunoreactive cells present in the gate were isolated from wild type mice (C, lower panel) and Sdc4^−/−^ mice (D, lower panel) where the percentages indicate satellite cells (blue lines) relative to other events with false-positives set to an antibody background < 0.1% (red lines). A hierarchical dendrogram constructed with Spotfire™ DecisionSite using Affymetrix GeneChip data reveals that Sdc4^−/−^ satellite cells cluster most closely to freshly isolated wild type satellite cells while injured wild type satellite cells either 12 h post-injury or 48 h post-injury cluster independently (**E**). Red depicts high relative gene expression and green depicts low relative expression in the hierarchical cluster dendrograms (UPGMA, Euclidean distance). FI, freshly isolated; PI, post-injury.

An unbiased global gene expression analysis using Affymetrix GeneChips was performed to identify changes occurring during the transition of satellite cells from quiescence to a population of proliferating myoblasts *in vivo*. To accomplish this, we identified changes in gene expression profiles between freshly isolated satellite cells and satellite cells isolated 12 h or 48 h following BaCl_2_-induced muscle injury. The time points chosen correspond to activated satellite cells which do not express MyoD protein (freshly isolated), committed myoblasts marked by MyoD expression (12 h post-muscle injury), and proliferating myoblasts (48 h post-muscle injury; Figure 1A) [36,37]. Syndecan-4 null satellite cells fail to activate, express MyoD, or enter the cell cycle appropriately within 48 h post-injury, and are incapable of skeletal muscle repair (Figure 1B) [36,37]. Therefore, we eliminated the genes whose expression changes following a muscle injury in Sdc4^−/−^ satellite cells from our analyses as these genes were unlikely to be involved in satellite cell activation. Wild type and Sdc4^−/−^ satellite cells were isolated by fluorescence activated cell sorting (FACS) using anti-syndecan-3 antibodies as a marker for quiescent and proliferating satellite cells [38] from uninjured tibialis anterior (TA) muscle (Figure 1C) and TA muscles 12 h post-injury (Figure 1D).

RNA was extracted from the isolated cells and processed for hybridization to Affymetrix v2.0 mouse GeneChips. The GeneChip data (Additional file 1, Additional file 2, Additional file 3, Additional file 4, Additional file 5, Additional file 6) were analyzed with Spotfire™ DecisionSite for Microarry Analysis software and an initial hierarchical cluster dendrogram generated. Visualization of the relationships between gene expression profiles show Sdc4^−/−^ samples, regardless of time post-injury, cluster with freshly isolated wild type satellite cells (Figure 1E) supporting our observations that Sdc4^−/−^ satellite cells do not activate appropriately within 48 h following an induced muscle injury (Figure 1A, B). Within the wild type dataset, we observed that committed myoblasts isolated 12 h post-injury exhibited the most divergent gene expression profiles, suggesting that these committed satellite cells differ substantially from either quiescent satellite cells or proliferating myoblasts (Figure 1E). To focus on genes that may be involved in satellite cell activation, we chose to further compare gene expression changes occurring within the first 12 h post-muscle injury.

### Over 4,000 genes are specifically regulated during satellite cell activation

**Figure 2:**
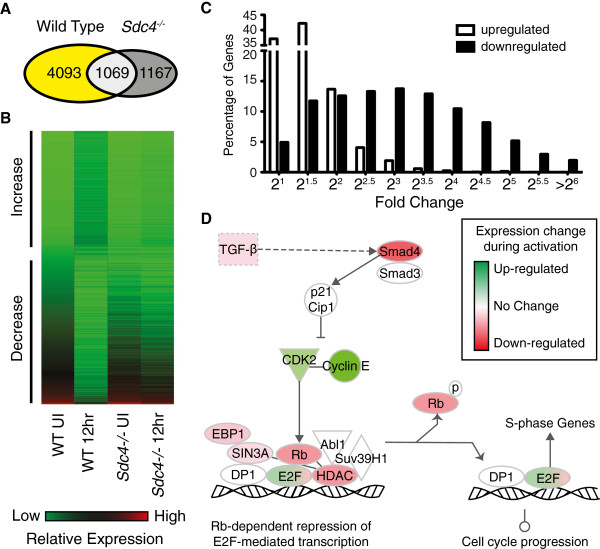
**Gene expression changes occurring during satellite cell activation.** The genes significantly regulated between freshly isolated satellite cells and satellite cells isolated 12 h post-injury from wild type and Sdc4^−/−^ mice were plotted as a Venn diagram to identify genes unique to wild type satellite cells (**A**, yellow, ANOVA *P* ≤ 0.01, ≥ two-fold change). A heat map depicting changes in relative expression of genes unique to wild type satellite cells with more than half (56%) of the transcripts decreasing during the first 12 h following satellite cell activation (**B**, red is high relative expression and green low relative expression). The frequency of genes that decrease (■) more than four-fold (2^2^) is significantly higher than the frequency of genes that increase (□) more than four-fold (2^2^) during the first 12 h post-muscle injury (**C**). Further analysis of gene expression data using IPA 9.0 (Ingenuity® Systems, http://www.ingenuity.com) demonstrate that genes promoting cell cycle progression increase (green) while genes that inhibit the G1/S phase transition decrease (red) in wild type satellite cells 12 h post-muscle injury (**D**, relative intensity depicts the fold change with higher color intensity denoting a greater fold change).

A comparative analysis of gene expression profiles from wild type and Sdc4^−/−^ satellite cells within the first 12 h following satellite cell activation identified a cohort of genes unique to satellite cell activation. In wild type satellite cells, 5,162 genes change significantly between satellite cells isolated from uninjured muscle and those isolated 12 h post-injury as defined by a ≥ 2-fold change with an ANOVA *P* ≤ 0.01 (Figure 2A). In contrast, 2,236 genes similarly changed expression in Sdc4^−/−^ satellite cells isolated from uninjured TA muscle and TA muscles 12 h post-injury (Figure 2A). Eighty percent (4,093) of the genes differentially expressed in WT satellite cells do not significantly change in Sdc4^−/−^ satellite cells as identified by Venn analysis (Figure 2A, B; Additional file 7). We reasoned that the metabolic changes occurring during satellite cell activation as well as the induction of the transcription factor MyoD and cell cycle entry would result in a large cohort of induced genes. Surprisingly, more than half (56%) of the genes dif ferentially expressed in satellite cells by 12 h post-injury *decrease* in relative expression (Figure 2B). Moreover, the magnitude of change for genes that decrease is on average three-fold greater than the magnitude of change for genes that increase following muscle injury. Less than 10% of genes whose expression is increased change more than four-fold (2^2^), while 70% of downregulated genes change more than four-fold (2^2^) and 3% decrease more than 64-fold (2^6^) (Figure 2C). These observations suggest that quiescent satellite cells express a cohort of genes that maintains and regulates the quiescent state, are likely critical for interaction with the satellite cell niche, and are necessary for interpreting signals for exit from quiescence. Furthermore, our results support the idea that satellite cell quiescence is actively maintained, consistent with a prior report examining freshly isolated satellite cells and satellite cells isolated from dystrophic muscle [14].

To further test the idea that satellite cell quiescence is actively maintained, we analyzed genes in the cohort that significantly change 12 h post-injury involved in cell cycle progression. We would expect cell cycle progression genes to be induced during activation and found that genes modulating the G1/S phase transition are among those that increase in relative expression (Figure 2D, green). In contrast, cell cycle inhibitors decrease in relative expression (Figure 2D, red) as expected for the transition of satellite cells from mitotic quiescence to an activated state in preparation for cell cycle entry. Moreover, these genes do not change expression significantly in Sdc4^−/−^ satellite cells 12 h post-injury (Figure 2A, B; Additional file 7) consistent with their impaired cell cycle activation and MyoD induction.

### Genes involved in RNA post-transcriptional regulation are significantly enriched during satellite cell activation

**Figure 3:**
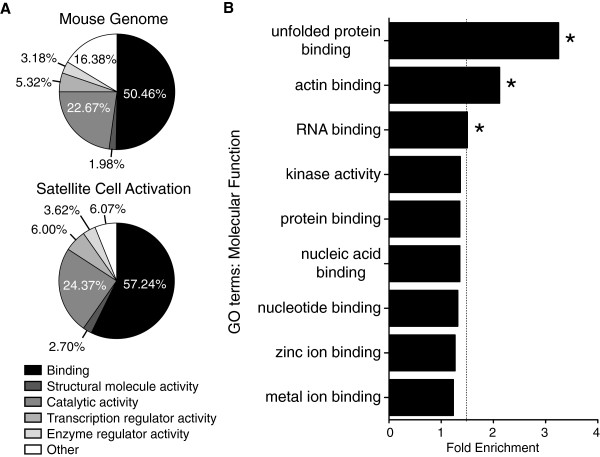
**Binding genes are enriched during satellite cell activation.** Gene expression changes unique to wild type satellite cells occurring within 12 h post-muscle injury were further analyzed by gene ontology. The general Molecular Function GO categories of Binding (*P* = 7.03 × 10^-43^), Structural molecule activity (*P* = 4.97 × 10^-4^), Catalytic activity (*P* = 1.46 × 10^-3^), Transcription regulator activity (*P* = 0.0229), and Enzyme regulator activity (*P* = 0.0521) were identified by DAVID as enriched when comparing satellite cells isolated 12 h post-muscle injury to freshly isolated satellite cells (**A**). Specific Molecular Function GO terms including RNA binding, unfolded protein binding, and actin binding were enriched an average of 1.5-fold when comparing satellite cells from injured and uninjured muscle as identified by three independent algorithms including ProfCom, FunNet, and DAVID (**B**). The dotted line marks a 1.5-fold enrichment threshold. Asterisks mark GO terms with an average enrichment ≥ 1.5-fold.

Gene ontology (GO) classifications were used to aid in identifying potential mechanisms regulating satellite cell activation. The Database for Annotation, Visualization and Integrated Discovery (DAVID) was used to identify enrichment of general molecular function categories [39,40] during activation of satellite cells. The GO category of Molecular Function: Binding is the most significantly over-represented GO category during satellite cell activation (*P* value = 7.03 × 10^-43^ compared to the mouse genome), where a 7% increase in the total percentage of genes classified as binding occurs in satellite cells within the first 12 h post-muscle injury (Figure 3A). Further refinement of ontological categories reveals that GO terms unfolded protein binding, actin binding, and RNA binding were enriched an average of 1.5-fold over three independent gene annotation algorithms: FunNet [41,42], ProfCom [43], and DAVID [39,40] (Figure 3B, Table 2). Thus, in the first 12 h post-muscle injury, major changes occur in genes involved in RNA binding, the unfolded protein response, and in actin binding. The changes in RNA binding proteins and unfolded protein response may be involved in the down-regulation of genes necessary to maintain a quiescent satellite cell, while changes in actin binding are likely to reflect changes in satellite cell motility [44] that accompany repair of skeletal muscle tissue.

**Table 2:** Identified Molecular Function GO terms

**GO Term**	**DAVID**	***P*value**	**ProfCom**	***P*value**	**FunNet**	***P*value**
Unfolded protein binding	2.27	2.74E-05	4.65	2.90E-08	2.83	2.64E-08
Actin binding	1.83	2.00E-09	2.60	2.10E-12	1.92	5.03E-10
RNA binding	1.30	3.25E-04	1.82	9.80E-10	1.39	6.98E-05
Kinase activity	1.25	7.15E-04	1.54	4.30E-08	1.31	1.48E-04
Protein binding	1.25	8.42E-29	1.52	5.90E-70	1.30	1.86E-21
Nucleic acid binding	1.13	2.38E-04	1.63	7.50E-10	1.31	7.34E-05
Nucleotide binding	1.21	6.45E-07	1.50	6.10E-18	1.23	2.76E-06
Zinc ion binding	1.19	1.83E-05	1.38	1.80E-15	1.23	5.01E-05
Metal ion binding	1.17	7.66E-09	1.37	5.20E-23	1.16	4.83E-05

Fold enrichment and associated p-values for the Molecular Function GO terms shared between three independent gene ontological analyses using DAVID (http://david.abcc.ncifcrf.gov), profcom (http://webclu.bio.wzw.tum.de/profcom/), and FunNet (http://www.funnet.info).

A fourth independent analysis focused on biological networks (IPA-Ingenuity Pathway Analysis http://www.ingenuity.com) ranked RNA Post-Transcriptional Modification in the top biological network (Table 3; Table 4). Thus, from four independent methods of gene expression analysis, a much higher proportion of genes involved in post-transcriptional RNA regulation change expression in the transition from a quiescent satellite cell to a committed myoblast, suggesting a role for post-transcriptional regulation of RNA in this transition. Therefore, we further analyzed genes involved in RNA post-transcriptional modification to further characterize individual genes and to develop hypotheses regarding the function of these genes in the transition of satellite cells from mitotic quiescence to cell cycle entry.

**Table 3:** Top 25 ranking biological interaction networks enriched during satellite cell activation

**Rank**	**Associated network function**	**Score**
1	RNA post-transcriptional modification, developmental disorder, genetic disorder	34
2	Genetic disorder, neurological disease, psychological disorders	34
3	Genetic disorder, cellular assembly and organization, skeletal and muscular disorders	34
4	Lipid metabolism, small molecule biochemistry, vitamin and mineral metabolism	32
5	Nervous system development and function, tissue morphology, cellular development	32
6	Genetic disorder, metabolic disease, molecular transport	32
7	Cell morphology, cell-to-cell signaling and interaction, cellular assembly and organization	32
8	Genetic disorder, ophthalmic disease, cardiovascular disease	32
9	Post-translational modification, cardiovascular disease, cardiovascular system development and function	32
10	Amino acid metabolism, genetic disorder, metabolic disease	32
11	Cardiovascular system development and function, cell morphology, cell-to-cell signaling and interaction	32
12	Dermatological diseases and conditions, genetic disorder, amino acid metabolism	32
13	Organismal functions, cardiac stenosis, cardiovascular disease	32
14	Cellular assembly and organization, RNA post-transcriptional modification, cancer	32
15	Carbohydrate metabolism, drug metabolism, nucleic acid metabolism	32
16	Gene expression, amino ccid metabolism, small molecule biochemistry	32
17	Cell cycle, reproductive system development and function, cell morphology	32
18	Genetic disorder, neurological disease, psychological disorders	32
19	Cancer, cellular assembly and organization, cellular compromise	32
20	Cell cycle, cell death, cell morphology	30
21	Genetic disorder, metabolic disease, neurological disease	30
22	Post-translational modification, protein degradation, protein synthesis	29
23	Cell signaling, cardiovascular disease, skeletal and muscular system development and function	29
24	Lipid metabolism, small molecule biochemistry, dermatological diseases and conditions	29
25	Cellular development, genetic disorder, hematological system development and function	29

The most significantly enriched biological networks during satellite cell activation were generated through the use of IPA 9.0 (Ingenuity Systems, http://www.ingenuity.com). The score is the negative base-10 logarithm of the *P* value (that is, a score of 34 is *P* ≤ 10^-34^).

**Table 4:** Genes in the top ranked network associate with muscle function, muscle disease, or fate determination

**Genes**	**Relevant role/Disease/Expression**	**Reference**
Luc7l	Regulation of muscle differentiation	[45]
Snrpn	Prader-Willi syndrome	[46]
Polr2a	Positive regulation of embryonic stem cells	[47]
Htatsf1	Expression in developing limb bud	[48]
Zbtb3	Expression in developing limb bud	[48]
Supt5h	Expression in developing limb bud	[48]
Tcerg1	Expression in developing limb bud	[48]
Pqbp1	Facilitates neuronal proliferation and maturation; Expression in developing limb bud	[48,49]
Snrpb	Spinal muscular atrophy; Expression in developing limb bud and somite	[7,48]
Snrpa1	Spinal muscular atrophy	[50]
Syncrip	Spinal muscular atrophy	[50]
Sf1	Expression in developing limb bud	[48]
AP3D1	Regulation of progenitor cell competence	[51]
Hnrnpr	Expression in developing limb bud	[48]

Fourteen of the thirty-five genes in the top ranked biological network as identified by IPA v9.0 (Ingenuity® Systems, http://www.ingenuity.com) have defined functions in muscle or cell fate determination as determined with the associated references.

### Splicing factors are preferentially downregulated during satellite cell activation

**Figure 4:**
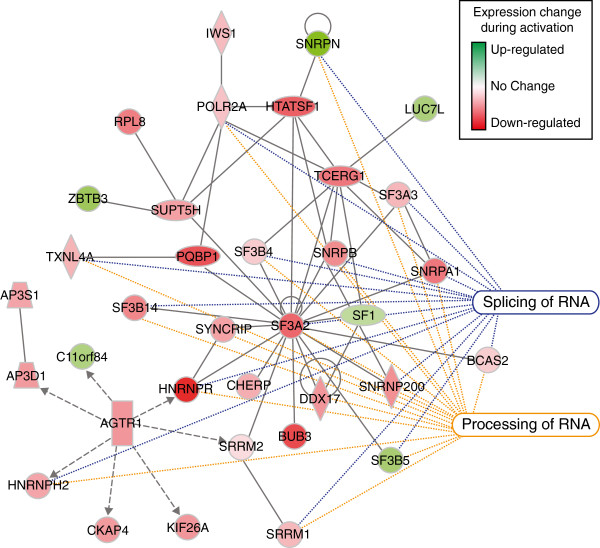
**RNA post-transcriptional modification is the most enriched biological network.** Gene expression changes unique to wild type satellite cell activation were subjected to IPA 9.0 (Ingenuity® Systems, http://www.ingenuity.com) network analysis and RNA Post-Transcriptional Modification, Developmental Disorder, Genetic Disorder (*P* ≤ 10^-34^) emerged as the associated biological functions in the top ranked interaction network with the canonical pathways Processing of RNA (17 blue dotted lines, *P* ≤ 4.44e-22) and Splicing of RNA (15 orange dotted lines, *P* ≤ 7.64e-20) comprising 50% of this network. The functions of these genes in muscle disease and cell fate decisions are listed in Table 4. Red indicates genes down-regulated and green indicates genes upregulated with the intensity denoting the increase or decrease in fold change. The data compares genes unique to wild type satellite cells isolated 12 h post-injury to satellite cells isolated from uninjured skeletal muscle.

**Figure 5:**
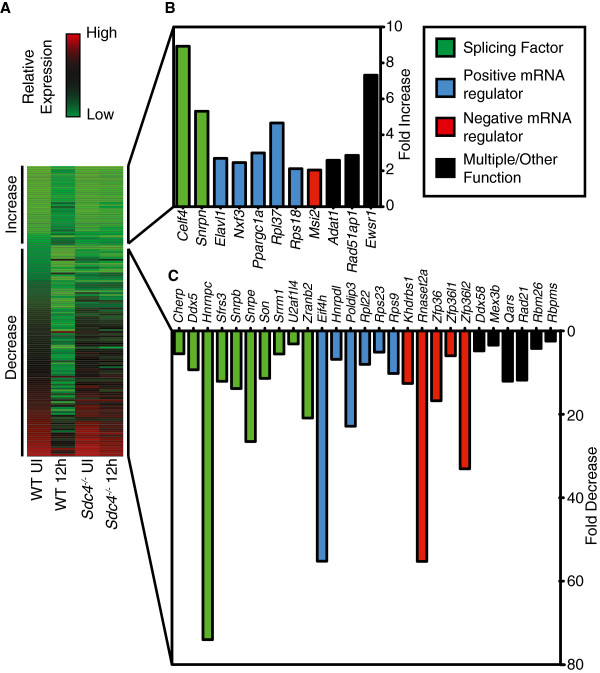
**Classification of top quartile RNA binding proteins significantly regulated 12 h post-injury in satellite cells.** Genes categorized with the GO term Molecular Function: RNA Binding that change expression ≥ two-fold (ANOVA *P* ≤ 0.01) comprise 22% (154 of 716) of the total GO category in wild type satellite cells but do not change significantly in Sdc4^−/−^ satellite cell during the first 12 h post-muscle injury (**A**). A minority of the identified genes increase in relative expression (**B**), while the majority of these genes decrease in their relative expression 12 h post-muscle injury (**C**). Upregulated and downregulated genes were further classified and plotted as splicing factors (green bars), positive mRNA regulators (blue bars), negative mRNA regulators (red bars), or multiple/other functions (black bars). The values plotted are for fold increase (**B**) or fold decrease (**C** )unique to wild type satellite cells occurring in the first 12 h post-muscle injury.

The top ranking biological network identified by IPA has associated biological functions of RNA Post-Transcriptional Modification, Developmental Disorder, and Genetic Disorder (Table 3), where RNA Post-Transcriptional Modification includes mRNA decay and stabilization, mRNA splicing, and miRNA-mediated gene silencing. When we examined genes within this network, nearly half of the genes whose expression changes during satellite cell activation are involved in RNA processing and splicing (Figure 4). Moreover, many genes in this interaction network are implicated in either muscle function, muscle disease, or cell fate decisions (Table 4). Consistent with our prior observations that downregulated genes exhibit greater fold change in relative expression occurring during the first 12 h post-muscle injury, the majority of RNA processing and splicing genes identified in the interaction network (87% and 88%, respectively) decrease post-muscle injury (Figure 4). Furthermore, of the 154 RNA binding proteins identified by GO analysis (Figure 3B, Additional file 8), 69% decrease in relative expression (Figure 5A; Additional file 8). These data show that RNA binding proteins are highly over-represented in quiescent satellite cells and suggest that regulation of RNA plays an important role in maintaining the quiescent state and in the transition to a cycling myoblast.

An examination of the top quartile of RNA binding proteins with the most significant changes in expression reveal that the majority (45%) of upregulated genes are positive mRNA regulators (Figure 5B), while downregulated genes include a similar distribution of all RNA protein functions with splicing factors exhibiting a slight majority (37%: Figure 5C). Furthermore, 24% of the previously identified RNA processing of splicing factors (Figure 4) are present in this top quartile. To confirm the microarray expression data, we performed qPCR validation finding that approximately 80% of the tested RNA binding proteins and both tested cell cycle regulators have consistent gene expression profiles (Table 5). Thus, genes involved in mRNA regulation may play diverse roles in satellite cell activation and promote the conversion of a quiescent satellite cell to a proliferating myoblast.

**Table 5:** Microarray and qPCR expression trends correlate for RNA binding proteins

**Gene**	**Microarray**	**qPCR**	**Fold change**	**Correlation**	**Biological function**
Celf4	↑	↑	2.51	Yes	Splicing factor
Pabpn1	↓	↓	−1.80	Yes	Positive mRNA regulator
Ppargc1a	↑	↑	1.34	Yes	Positive mRNA regulator
Mbnl1	↓	↓	−1.14	Yes	Splicing factor
Matr3	↓	↑	11.79	No	Negative mRNA regulator
Sfrs3	↓	↑	19.11	No	Splicing factor
Zfp36	↓	↓	−2.87	Yes	Negative mRNA regulator
Zfp36l1	↓	↓	−4.48	Yes	Negative mRNA regulator
Zfp36l2	↓	↓	−1.63	Yes	Negative mRNA regulator
Elavl1	↑	↑	3.01	Yes	Positive mRNA regulator
Cdk2	↑	↑	10.28	Yes	Promotes cell cycle entry
E2F3	↑	↑	12.90	Yes	Promotes cell cycle entry

Eight of ten RNA binding proteins and two cell cycle genes validate expression changes between wild type satellite cells isolated from uninjured TA muscle and from the TA 12 h post-muscle injury. Arrows show increase (↑) and decrease (↓) for both microarray and qPCR during satellite cell activation. Fold change is from qPCR data where positive values indicate increased expression and negative valued indicate decreased expression between satellite cells isolated from uninjured TA muscle and from the TA 12 h post-muscle injury. Quantitative PCR data is normalized to GAPDH or 18S.

### Dynamic regulation of miRNAs during muscle regeneration

MicroRNA-mediated gene silencing regulates alternative splicing [52,53] as well as mRNA stability factors [54,55]. Moreover, miRNAs regulate stem cell fate determination [28,30,31,56] suggesting a potential role for miRNAs in the transition of quiescent satellite cells to proliferating myoblasts. The low levels of RNA present in quiescent satellite cells combined with infrequent satellite cell abundance in uninjured muscle prevented successful unbiased screen for miRNAs. Therefore, we assessed whether genes involved in miRNA biogenesis and gene silencing including argonautes1-4 (Eif2c1-4), Dgcr8, and Dicer, as well as other genes associated with miRNA function, are expressed in quiescent satellite cell (Additional file 1, Additional file 2, Additional file 3, Additional file 4, Additional file 5, Additional file 6). Although present, Argonautes1-4 (Eif2c1-4), Dgcr8, and Dicer are not in the cohort of differentially expressed genes (Additional file 7), suggesting that major changes in miRNA processing do not occur during satellite cell activation.

**Figure 6:**
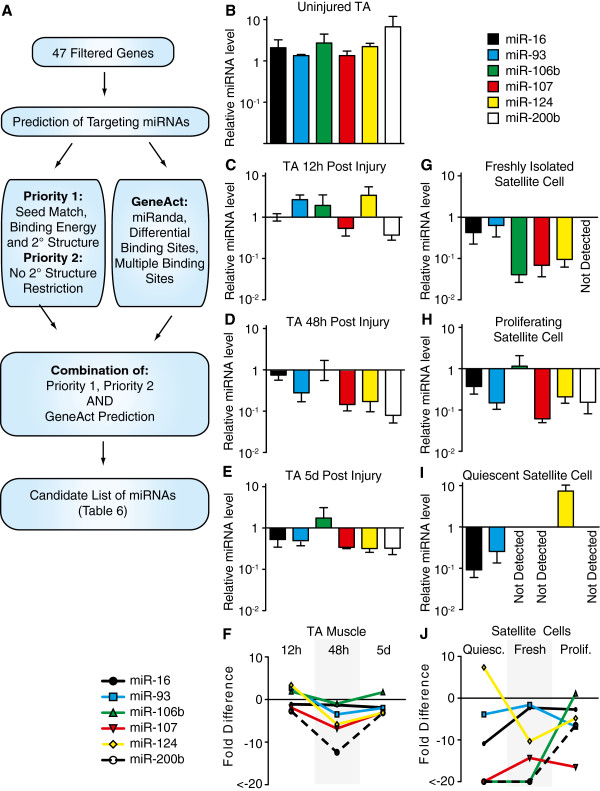
**Screening and characterization of candidate miRNAs with dynamic expression patterns during muscle regeneration.** A combinatorial screen was used to identify miRNAs from potential target genes that uniquely change expression in wild type satellite cells following a muscle injury. Gene expression changes of ≥ two-fold, *P* ≤ 0.01 occurring in wild type satellite cells 48 h post-muscle injury compared to freshly isolated satellite cells that changed ≤ two.0-fold, *P* ≥ 0.9 in syndecan-4 null cells yielded 47 genes (Table 6) that were subjected to the flow schematic to identify potential miRNAs (**A**). The union from both algorithms yielded 12 candidate miRNAs, six of which were detectable by RNase protection assay in cultured satellite cells or the satellite cell derived MM14 cell line in growth or differentiation conditions (Table 7). These six miRNAs are expressed in uninjured skeletal muscle **(B)** and four of the six change expression dynamically during skeletal muscle regeneration at 12 h (**C**, **F**), 48 h (**D**, **F**) and 5d (**E**, **F**) post-muscle injury. The same four micro RNAs (miR-16, miR-93, miR-106b, and miR-124) exhibit dynamic changes in relative expression when comparing activated satellite cells (**G**, **J**) to proliferating satellite cells (**H**, **J**) and quiescent satellite cells (**I**, **J**). All qPCR data was normalized to U6 RNA. miRNA levels in uninjured TA muscle were set to 1 (y-axis). Values above the y-axis indicate higher miRNA expression than in uninjured TA muscle and values below the y-axis indicate lower miRNA expression than in uninjured TA muscle (**C**-**J**). Graphs B-E and G-I are log scale and values are mean ± SEM (*n* = 3). Graphs F and J are average fold difference as compared to relative expression in B.

To identify potential miRNAs involved in the transition of quiescent satellite cells to proliferating myoblasts, we applied miRNA target prediction algorithms to identify putative miRNAs regulating genes whose expression changes rapidly during the first 48 h post-muscle injury. Initially, we established a minimum gene expression value in freshly isolated satellite cells to reduce the cohort to 641 genes (ANOVA *P* ≤ 0.01) that either increased or decreased by ≥ two-fold during the first 48 h following muscle injury. As gene expression levels did not change for 47 of these genes in Sdc4^−/−^ satellite cells (ANOVA *P* ≥ 0.9), we chose these 47 genes to pursue as potential miRNA targets involved in satellite cell activation (Table 6). Candidate miRNAs were then computationally identified using two independent algorithms, one developed by the group of Deepak Srivastava (unpublished) and GeneAct (http://promoter.colorado.edu/geneact/) [57] (Figure 6A). The union of both algorithms identified 12 miRNA candidates with six, miR-16, miR-93, miR-106b, miR-107, miR-124, and miR-200b, being detected in cultured primary satellite cells or proliferating satellite cell derived MM14 cells by ribonuclease protection assay (Table 7). All six miRNAs present in primary myoblasts and MM14 cells were detectable in uninjured tibialis anterior muscle (Figure 6B).

**Table 6:** Genes used to predict candidate miRNAs

**Probe set ID**	**Gene symbol**	**Entrez gene**	**Fold change**
1417654_at	Sdc4	20971	11.68
1418282_x_at	Serpina1b	20701	6.78
1418510_s_at	Fbxo8	50753	4.74
1419070_at	Cys1	12879	3.78
1419302_at	Heyl	56198	3.26
1420930_s_at	Ctnnal1	54366	2.71
1420980_at	Pak1	18479	3.13
1422889_at	Pcdh18	73173	4.72
1422892_s_at	H2-Ea	14968	25.61
1424559_at	Rpap2	231571	5.15
1425336_x_at	H2-K1	14972	34.35
1425609_at	Ncf1	17969	2.68
1426981_at	Pcsk6	18553	3.38
1427884_at	Col3a1	12825	12.29
1429021_at	Epha4	13838	3.51
1430764_at	1700023F06Rik	69441	3.12
1433639_at	5730593F17Rik	215512	3.30
1434105_at	Epm2aip1	77781	2.91
1434267_at	Nek1	18004	2.90
1434790_a_at	Lta4h	16993	4.96
1435603_at	Sned1	208777	3.12
1437152_at	Mex3b	108797	4.08
1438532_at	Hmcn1	545370	6.09
1438577_at	---	---	5.66
1439618_at	Pde10a	23984	3.37
1440237_at	Ercc4	50505	3.40
1441958_s_at	Ager	11596	7.06
1442700_at	Pde4b	18578	11.21
1444409_at	Rph3al	380714	3.92
1444517_at	---	---	3.13
1447257_at	---	---	2.45
1447657_s_at	Synpo2l	68760	3.00
1449226_at	Hic1	15248	3.11
1449465_at	Reln	19699	5.95
1449619_s_at	Arhgap9	216445	3.20
1451513_x_at	Serpina1a	20700	4.62
1452632_at	Aak1	269774	3.64
1452896_at	Gtl3	14894	8.40
1453114_at	Nol9	74035	2.30
1453771_at	Gulp1	70676	5.06
1454112_a_at	Cep27	66296	2.46
1454433_at	6330526H18Rik	76174	2.95
1454877_at	Sertad4	214791	6.96
1455136_at	Atp1a2	98660	6.60
1455188_at	Ephb1	270190	3.37
1457944_at	---	---	16.23
1459164_at	AU014678	101228	2.34

The relative expression data for genes that significantly change (ANOVA *P* ≤ 0.01, ≥ two-fold change) in wild type satellite cells isolated from uninjured TA muscle or from the TA 48 h post-muscle injury and not in Sdc4^−/−^ satellite cells (ANOVA *P* > 0.9) is represented as a log_2_ values. Gene identifiers are Probe set ID, representative gene symbol, and entrez gene ID. The fold change was calculated for changes occurring in the first 12 h post-muscle injury according to genotype.

**Table 7:** Six of twelve predicted miRNAs are expressed in satellite cells

**miRNA**	**Rationale**	**Detected in SCs**
miR-16	Priority 2 and GeneAct	+
miR-26a/b	Priority 1 and GeneAct	ND
miR-30a	Priority 1, 2 and GeneAct	-
miR-93	Priority 1 and GeneAct	+
miR-106b	Priority 1 and GeneAct	+
miR-107	Priority 2 and GeneAct	+
miR-124	Priority 1 and GeneAct	+
miR-130a	Priority 1 and GeneAct	-
miR-132	Priority 1 and GeneAct	-
miR-200b	Priority 1 and GeneAct	+
miR-320	Priority 1 and GeneAct	-
miR-424	Priority 1 and GeneAct	-

We predicted 12 candidate miRNAs from 47 genes identified as only expressed in satellite cells or only expressed in myoblasts. miR-16, miR-93, miR-106b, miR-107, miR-124, and miR-200b are detected in satellite cells by RNase protection assay in either primary satellite cells or in the satellite cell derived MM14 cell line.

We observed dramatic regulation of these six miRNAs following a muscle injury when compared to control, uninjured tibialis anterior muscle. Four of the identified miRNAs (miR-93, miR-107, miR-124, and miR-200b) changed expression levels by more than two-fold during the first 5 days following induced muscle injury (Figure 6B-F; Table 8). In the uninjured TA muscle, miR-200b decreased three-fold by 12 h post-injury, while miR-93 and miR-124 increased significantly 12 h post-muscle injury (Figure 6B, C, F; Table 8). Within 48 h post-injury, the relative levels of miR-93, miR-107, and miR-124 had decreased levels well below those present in uninjured muscle and remained low at 5 days post-injury (Figure 6C-E). In contrast, miR-106b remained elevated following injury while miR-16 trended to slightly lower expression (Figure 6B-E). The rapid changes in miRNA relative expression and their presence in skeletal muscle suggest that these miRNAs may play important roles in the regeneration of skeletal muscle and validates our approach to identify such miRNAs.

**Table 8:** Fold difference of candidate miRNAs as compared to levels in resting muscle

	**miR-16**	**miR-93**	**miR-106b**	**miR-107**	**miR-124**	**miR-200b**
12 h PI	−1.1	2.7	1.9	−1.9	3.4	−2.7
48 h PI	−1.3	−3.5	NC	−6.8	−5.8	−12.4
5d PI	−1.9	−2.0	1.7	−3.0	−3.1	−3.1
Quiescent satellite cell	−10.9	−3.9	ND	ND	7.4	ND
Freshly isolated satellite cell	−2.3	−1.6	−24.3	−14.3	−10.3	−1418.2
Proliferating satellite cell	−2.7	−6.8	1.1	−16.5	−4.8	−6.5

Fold difference of miRNA expression as compared to resting muscle where positive values are higher expression and negative values are lower expression. NC, no change; ND, not detected.

### Relative expression of miRNAs in satellite cells following muscle injury

We asked whether the six miRNAs that change expression during muscle regeneration are present in satellite cells, muscle tissue, or both. The relative expression levels for each miRNA in the uninjured tibialis anterior muscle was normalized to 1 and the relative levels in satellite cells isolated from uninjured muscle and in proliferating satellite cells isolated 48 h post-injury examined. Surprisingly, all six miRNAs were expressed at low to undetectable levels in freshly isolated satellite cells (Figure 6G). In proliferating satellite cells, all but miR-106b were expressed at levels substantially lower than that found in the tibialis anterior muscle (Figure 6H). Although MyoD protein is not detectable in freshly isolated satellite cells and they have not yet entered S-phase, freshly isolated satellite cells are not quiescent since the p38α/β MAPK is activated [8]. Therefore, to identify miRNAs present in quiescent satellite cells, mice were injected with SB203580, a p38α/β MAPK inhibitor, 1.5 h prior to satellite cell isolation (Hausburg *et al*., Submitted), and relative miRNA levels examined. Remarkably, we found that miR-124 was expressed 35-fold higher in quiescent satellite cells than in freshly isolated or proliferating satellite cells (Figure 6I, J; Table 8). Moreover, miR-124 was expressed at levels 10-fold greater in quiescent satellite cells than in uninjured skeletal muscle (compare Figure 6I and 6B, Figure 6J; Table 8), suggesting that the primary source of miR-124 in uninjured muscle is the satellite cell population. In contrast to miR-124, miR-16 and miR-93 are present at low to undetectable levels in quiescent satellite cells and are induced in freshly isolated satellite cells (Figure 6G, I). The expression level of miR-16 is maintained in proliferating cells, while miR-93 declines and miR-106b is dramatically induced in proliferating satellite cells as compared to freshly isolated and quiescent satellite cells (Figure 6G-I).

### miR-16, miR-106b, and miR-124 regulate satellite cell fate

**Figure 7:**
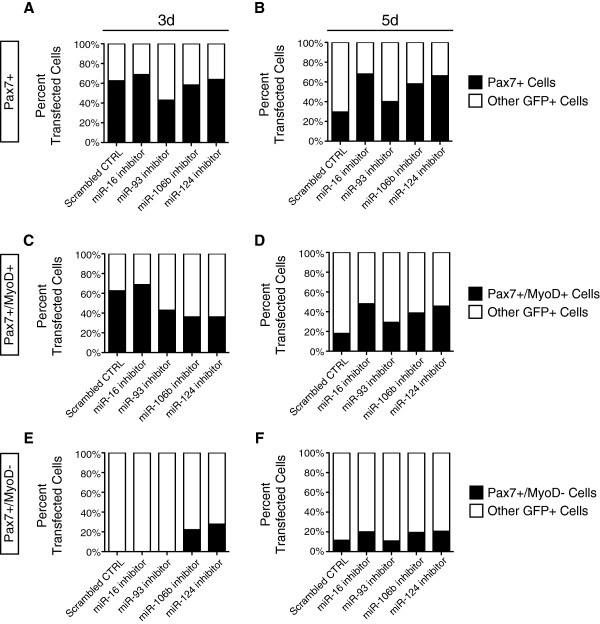
**Inhibition of candidate miRNAs alters satellite cell fate.** The four candidate miRNAs (miR-16, miR-93, miR-106b, and miR-124) that displayed dynamic expression in satellite cells were inhibited in myofiber-associated satellite cells prior to the first cell division. Transfected cells were assessed 3 or 5 days post myofiber harvest and identified via immunofluorescence as satellite cells by Pax7 expression (**A**, **B**) with proliferating satellite cells expressing both Pax7 and MyoD (**C**, **D**) and quiescent satellite cells expressing only Pax7 (**E**, **F**). The percent of Pax7+ satellite cells decreased between 3 and 5 days in satellite cell populations treated with a scrambled RNA control, however, the relative number of Pax7+ satellite cells remained at similar levels when any candidate miRNA was inhibited (**A**, **B**). This increase in satellite cells following miRNA inhibition at 5 days was observed in both proliferating satellite cells (**D**) and quiescent satellite cells (**F**) for miR-16, miR-106b, and miR-124 while inhibition of miR-93 resulted in a specific increase in proliferating satellite cells at 5 days (**D**). Inhibition of two miRNAs, miR-106b and miR-124, resulted in a dramatic increase in quiescent satellite cells by 3 days post myofiber isolation (**E**) that remains consistent through 5 days post isolation (**F**).

**Figure 8:**
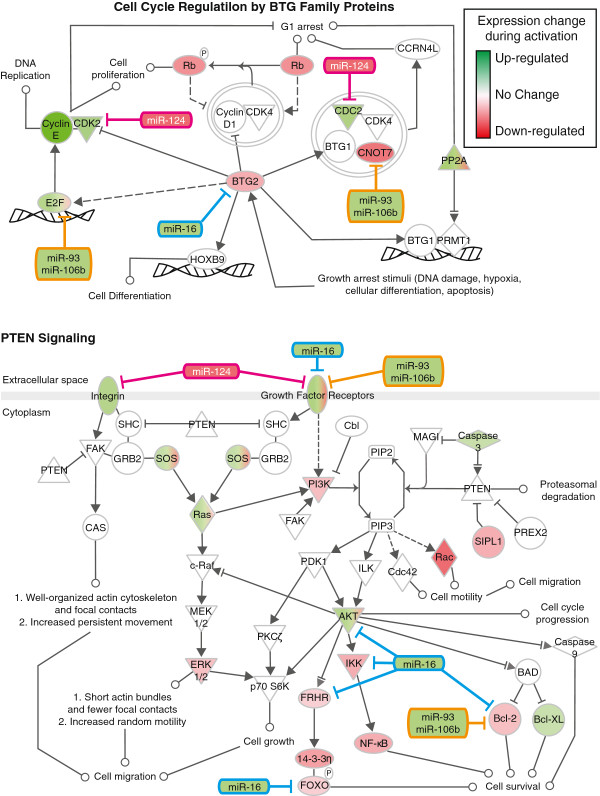
**Candidate miRNAs target genes involved in cell growth, survival, migration, and cell cycle progression.** The predicted target genes of miR-16, miR-93, miR-106b, and miR-124 were identified using Ingenuity® Systems (http://www.ingenuity.com). PTEN Signaling and Cell Cycle Regulation by BTG Family Proteins emerged as the top ranked canonical pathway regulated during satellite cell activation. The pathways and relative expression changes occurring during satellite cell activation are depicted. Note that the miRNAs have opposite expression profiles of their respective target mRNAs. Red indicates mRNAs downregulated and green indicates mRNAs upregulated with the intensity of red or green indicating increasing or decreasing fold change, respectively. The data compare mRNAs unique to wild type satellite cells isolated 12 h post-injury to satellite cells isolated from uninjured skeletal muscle.

The changes in relative levels of miR-16, miR-93, miR-106b, and miR-124 in satellite cells following a muscle injury suggests that these four miRNAs may play a role in the transition from a quiescent satellite cell to a proliferating myoblast. To test this idea, inhibitors for each miRNA were transfected into myofiber-associated satellite cells immediately following isolation and the cultures fixed and assayed at 3 days post-isolation and 5 days post-isolation. The total number of Pax7+ cells transfected with the scrambled RNA control inhibitor declined two-fold between 3 and 5 days in culture, indicative of differentiation (Figure 7A, B). Between 3 and 5 days in culture, the Pax7+/MyoD + decreased three-fold accompanied by the appearance of the Pax7+/MyoD- ‘reserve’ population (Figure 7C-F). In contrast, inhibition of miR-124 increased the percentage of Pax7+/MyoD- ‘reserve’ cells at 3 and 5 days of culture, as did inhibition of miR-106b (Figure 7A-F). Inhibition of miR-16 elevated the total number of Pax7+ cells at 5 days of culture and inhibition of miR-93 did not have any detectable effect (Figure 7-F). We further examined the role of miRNAs in satellite cell activation using Ingenuity® System’s IPA and identified PTEN signaling and Cell Cycle Regulation by BTG Family Proteins as the top canonical pathway regulated by miR-16, miR-93, miR-106b, and miR-124 in the transition of a quiescent satellite cell to a proliferating myoblasts (Figure 8). Many predicted targets of miR-16 and the miR-93/106b family inhibit cell cycle progression and cell growth. These targets are downregulated during satellite cell activation, consistent with increased expression of miR-16, miR-93, and miR-106b in proliferating satellite cells as compared to quiescent satellite cells (Figure 8). Conversely, predicted target genes of miR-124 promote cell cycle progression and are upregulated during satellite cell activation when miR-124 is downregulated (Figure 8). These data demonstrate that a number of miRNAs regulate satellite cell fate following a muscle injury and support the idea that post-transcriptional regulation of RNA plays a critical role in satellite cell activation and maintenance of satellite cell quiescence.

## Discussion

The low cytoplasmic to nuclear ratio, low organelle number, and mitotic quiescence of resident satellite cells [12,13] lead to the speculation that metabolic activity in these cells is low. Indeed, quiescent satellite cells with high levels of Pax7 express reduced levels of mitochondrial genes [58]. Moreover, the delay to the first cell cycle division (Troy *et al*.)^a^ coupled with the dramatic increase in cell size and mobility [36,44] suggests that satellite cell activation and cell cycle entry would require transcriptional induction of a large cohort of genes similar to that observed in serum stimulated fibroblasts [59]. However, we and others have postulated that quiescent satellite cells are poised for activation awaiting a critical signaling event [8,14]. The data presented here further support this hypothesis and provide the first direct comparison of quiescent satellite cells with activated satellite cells and proliferating myoblasts derived from uninjured and injured skeletal muscle, respectively. The prior analyses performed compared freshly isolated satellite cells with satellite cells isolated from dystrophic mice [14] or cultured cells [14,34] and are expected to identify gene expression changes associated with a diseased environment or a culture environment, respectively. Since the most significant reductions in gene expression occur within the first 12 h post-muscle injury, the metabolic and signaling events in a quiescent satellite cell are thus predicted to be divergent from those of a proliferating myoblast. Moreover, these data suggest that comparisons of freshly isolated satellite cells with proliferating myoblasts may not identify critical regulatory mechanisms involved in satellite cell activation [14,34].

Here, we used computational methods to initially identify that RNA post-transcriptional mechanisms are likely to maintain the quiescent satellite cell phenotype and to promote the conversion of the quiescent satellite cell to a transit-amplifying myoblast. Recent studies indicate that RNA post-transcriptional mechanisms, specifically alternative splicing, mRNA stability, and miRNA-mediated gene silencing, regulate stem cell pluripotency and progression through differentiation [17-20]. As satellite cells are an adult stem cell population, similar mechanisms may mediate the transition from quiescence to a population of proliferating myoblasts. We found that splicing factors may play roles in regulating the transition of satellite cells from quiescence to proliferating myoblasts. Consistent with these observations, the relative molar ratios of splicing factors guide alternative splicing [60] and these factors often function combinatorially to direct the expression of different mRNA splice variants [61]. Together with published data, our observations of dynamic splicing factor expression in satellite cells following muscle injury suggests that unique cohorts of mRNA species regulate the conversion of quiescent adult stem cells to the committed proliferating myoblast.

In addition to splicing factors, we found that mRNA binding proteins regulating mRNA stabilization and mRNA decay are preferentially upregulated in satellite cells following muscle injury. Indeed, the AU-rich element (ARE) binding protein HuR (Elavl1) is reported to stabilize MyoD and myogenin mRNA in skeletal muscle cell lines derived from satellite cells [62,63] potentially participating in satellite cell activation and commitment to myogenesis. Elavl1 is one of the most significantly upregulated RNA binding genes in satellite cells within 12 h of muscle injury (See Figure 5C; Additional file 8) and corresponds to MyoD expression. Interestingly, the ARE-binding proteins, tristetraprolin (Zfp36) and family members Zfp36l1 and Zfp36l2, decrease dramatically during the conversion from quiescence to activated satellite cells within the first 12 h post-muscle injury (See Figure 5D; Additional file 8). However, tristetraprolin and HuR have opposing functions as they counter-regulate expression of the same mRNAs [64,65] and may act as an agonist–antagonist pair for many genes that promote commitment to myogenesis. In agreement with these data, we have demonstrated that the Zfp36 family directly targets MyoD mRNA and functions to regulate satellite cell fate during satellite cell activation and self-renewal (Hausburg *et al*., Submitted).

A recent report identifies miR-489 as an important miRNA maintaining satellite cell quiescence [32] suggesting that miRNA-mediated gene silencing functions in the transition of a quiescent satellite cell to a proliferating myoblast. We identified a cohort of genes that significantly change expression in satellite cells within the first 48 h following muscle injury to computationally predict cognate miRNAs that may regulate these targets with two independent prediction algorithms. Six miRNAs not previously reported in skeletal muscle were selected for further analysis and all six were observed to be dynamically regulated in relative levels during induced muscle injury. Moreover, four of the six miRNAs were expressed in satellite cells (miR-16, miR-93, miR-106b, and miR-124), while two were likely present only in differentiated muscle (miR-107 and miR-200b). Comparing relative levels in muscle tissue and satellite cells revealed that miR-124 is likely only expressed in satellite cells, while miR-16, miR-93, and miR-106b are most likely expressed in satellite cells and in differentiated muscle fibers. Pathways predicted to be targeted by these miRNAs include cell cycle progression as well as PTEN signaling, which is involved in stem cell self-renewal [66] and muscle regeneration [67].

To directly test whether these four miRNAs regulate satellite cell behavior, we transfected inhibitors for each miRNA into myofiber-associated satellite cells shortly after isolation and examined the effects on satellite cell fate at 3 and 5 days post-myofiber isolation. Inhibition of two miRNAs, miR-106b and miR-124, increased the relative number of progenitor or ‘reserve’ satellite cells (Pax7+/MyoD-) relative to a control, suggesting that these miRNAs participate in the regulation of satellite cell fate and satellite cell self-renewal. In contrast, miR-16 enhanced the relative numbers of Pax7+ cells but did not appear to alter the percentage of Pax7+/MyoD + myoblasts or Pax7+/MyoD- ‘reserve’ cells relative to a scrambled control. Of the four miRNAs tested, the loss of miR-93 elicited no detectable changes in the numbers of ‘reserve’ Pax7+/MyoD- satellite cells and Pax7+/MyoD + satellite cells suggesting functions in cellular processes other than cell fate determination.

## Conclusions

We believe that RNA post-transcriptional regulation plays a critical role in the transition of a quiescent satellite cell to a transit-amplifying myoblast. At each time point we examined, including quiescent satellite cells (freshly isolated in the presence of a p38α/β MAPK inhibitor), activated satellite cells (12 h post-muscle injury), and proliferating myoblasts (48 h post-muscle injury), we found extensive changes in genes involved in post-transcriptional RNA regulation, including mRNA splicing, mRNA stability, and miRNA-mediated gene silencing. We conclude that satellite cell quiescence is actively maintained via combinatorial contributions primarily mediated through post transcriptional mRNA regulation and identified four miRNAs that likely play a role in the conversion of quiescent satellite cells to proliferating myoblasts.

## Endnotes

Following submission of our manuscript, the following was published demonstrating post-transcriptional regulation of myf-5 during satellite cells activation. Crist CG, Montarras D, Buckingham M (2012) Muscle Satellite Cells Are Primed for Myogenesis but Maintain Quiescence with Sequestration of Myf5 mRNA Targeted by microRNA-31 in mRNP Granules. Cell Stem Cell 11: 118-126.

## Abbreviations

ARE: AU-rich element; GO: gene ontology; PI: post injury; TA: tibialis anterior; UI: uninjured; WT: wild type.

## Competing interests

The authors do not have any competing interests.

## Authors’ contributions

NHF carried out bioinformatic analyses of the microarray data, designed and carried out the miRNA studies, and drafted the manuscript. MH carried out qRT-PCR for mRNA expression and many of the bioinformatic analyses of the microarray data. NDB carried out qRT-PCR for miRNA expression. CP carried out qRT-PCR for mRNA expression. DS carried out miRNA prediction. DDWC carried out satellite cell isolation and hybridization for the microarray. BBO conceived of the study, participated in its design and coordination, and helped to draft the manuscript. All authors read and approved the final manuscript.

## Supplementary Material

Additional file 1 **CEL files of wild type satellite cells isolated from uninjured muscle.** Raw expression data for three replicates of satellite cells isolated from wild type uninjured TA muscles. *. CEL files can be opened with any microarray analysis software including the Affymetrix Expression Console Software (http://www.affymetrix.com/estore/browse/level_seven_software_products_only.jsp?productId=131414&categoryId=35623&productName=Affymetrix-Expression-Console-Software#1_1). Clidk here for file

Additional file 2 **CEL files of wild type satellite cells isolated 12 h post-injury.** Raw expression data for three replicates of satellite cells isolated from wild type TA muscles 12 h post-injury. *. CEL files can be opened with any microarray analysis software including the Affymetrix Expression Console Software (http://www.affymetrix.com/estore/browse/level_seven_software_products_only.jsp?productId=131414&categoryId=35623&productName=Affymetrix-Expression-Console-Software#1_1). Clidk here for file

Additional file 3 **CEL files of wild type satellite cells isolated 48 h post-injury.** Raw expression data for three replicates of satellite cells isolated from wild type TA muscles 12 h post-injury. *. CEL files can be opened with any microarray analysis software including the Affymetrix Expression Console Software (http://www.affymetrix.com/estore/browse/level_seven_software_products_only.jsp?productId=131414&categoryId=35623&productName=Affymetrix-Expression-Console-Software#1_1). Clidk here for file

Additional file 4 **CEL files of syndecan-4**^**−/− **^**satellite cells isolated from uninjured muscle.** Raw expression data for three replicates of satellite cells isolated from syndecan-4^−/−^ uninjured TA muscles. *. CEL files can be opened with any microarray analysis software including the Affymetrix Expression Console Software (http://www.affymetrix.com/estore/browse/level_seven_software_products_only.jsp?productId=131414&categoryId=35623&productName=Affymetrix-Expression-Console-Software#1_1). Clidk here for file

Additional file 5 **CEL files of syndecan-4**^**−/− **^**satellite cells isolated 12 h post-injury.** Raw expression data for three replicates of satellite cells isolated from syndecan-4^−/−^ TA muscles 12 h post-injury. *. CEL files can be opened with any microarray analysis software including the Affymetrix Expression Console Software (http://www.affymetrix.com/estore/browse/level_seven_software_products_only.jsp?productId=131414&categoryId=35623&productName=Affymetrix-Expression-Console-Software#1_1). Clidk here for file

Additional file 6 **CEL files of syndecan-4**^**−/− **^**satellite cells isolated 48 h post-injury.** Raw expression data for three replicates of satellite cells isolated from syndecan-4^−/−^ TA muscles 12 h post-injury. *. CEL files can be opened with any microarray analysis software including the Affymetrix Expression Console Software (http://www.affymetrix.com/estore/browse/level_seven_software_products_only.jsp?productId=131414&categoryId=35623&productName=Affymetrix-Expression-Console-Software#1_1). Clidk here for file

Additional file 7 Satellite cell activation gene expression profile. Excel spreadsheet (*.xlsx) with columns for probe set ID, log_**2 **_**expression data, ANOVA *P *value, fold change, and gene ID for unique genes that significantly change (ANOVA *P * ≤ 0.01, ≥ two-fold change) in WT and not in Sdc4**^**−/− **^**satellite cells 12 h after BaCl**_**2 **_**-induced injury.** The ANOVA *P* value and fold change was calculated for changes occurring in the first 12 h post-muscle injury according to genotype. Clidk here for file

Additional file 8 **Molecular Function: RNA Binding genes regulated during satellite cell activation.** Excel spreadsheet (*.xlsx) of RNA binding proteins from Additional file 7 with gene identifiers of Probe set ID, representative gene symbol, and entrez gene ID. The relative expression data for genes that significantly change (ANOVA *P* ≤ 0.01, ≥ two-fold change) in wild type and in Sdc4^−/−^ satellite cells isolated from uninjured TA muscle or from the TA 12 h post-muscle injury is represented as a log_2_. Clidk here for file
